# Identifying Stable Electrocatalysts Initialized by Data Mining: Sb_2_WO_6_ for Oxygen Reduction

**DOI:** 10.1002/advs.202305630

**Published:** 2023-12-07

**Authors:** Xue Jia, Zixun Yu, Fangzhou Liu, Heng Liu, Di Zhang, Egon Campos dos Santos, Hao Zheng, Yusuke Hashimoto, Yuan Chen, Li Wei, Hao Li

**Affiliations:** ^1^ Advanced Institute for Materials Research (WPI‐AIMR) Tohoku University Sendai 980‐8577 Japan; ^2^ School of Chemical and Biomolecule Engineering The University of Sydney Darlington NSW 2006 Australia; ^3^ State Key Laboratory of Mechanical System and Vibration Shanghai Jiao Tong University Shanghai 200240 P. R. China; ^4^ Tohoku Forum for Creativity Tohoku University Sendai 980‐8577 Japan

**Keywords:** aqueous stability, data mining, electrocatalysts, electrochemistry‐induced surface stability, metal oxides, pH‐dependent microkinetic modeling

## Abstract

Data mining from computational materials database has become a popular strategy to identify unexplored catalysts. Herein, the opportunities and challenges of this strategy are analyzed by investigating a discrepancy between data mining and experiments in identifying low‐cost metal oxide (MO) electrocatalysts. Based on a search engine capable of identifying stable MOs at the pH and potentials of interest, a series of MO electrocatalysts is identified as potential candidates for various reactions. Sb_2_WO_6_ attracted the attention among the identified stable MOs in acid. Based on the aqueous stability diagram, Sb_2_WO_6_ is stable under oxygen reduction reaction (ORR) in acidic media but rather unstable under high‐pH ORR conditions. However, this contradicts to the subsequent experimental observation in alkaline ORR conditions. Based on the post‐catalysis characterizations, surface state analysis, and an advanced pH‐field coupled microkinetic modeling, it is found that the Sb_2_WO_6_ surface will undergo electrochemical passivation under ORR potentials and form a stable and 4*e*‐ORR active surface. The results presented here suggest that though data mining is promising for exploring electrocatalysts, a refined strategy needs to be further developed by considering the electrochemistry‐induced surface stability and activity.

## Graphical abstract

Initialized by the data mining from a bulk materials database, precious‐metal‐free Sb_2_WO_6_ is found to be a highly stable and active electrocatalyst for oxygen reduction reaction (ORR). Electrochemical surface state analysis and advanced pH‐dependent microkinetic modeling provide new insights into the high stability and performance of Sb_2_WO_6_ under ORR conditions.

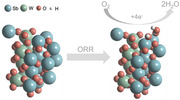

## 1 Introduction

The excessive extraction and processing of fossil fuels have caused serious impacts on the environment and natural resources, making it urgent to develop renewable energy storage and conversion technology to sufficiently use renewable energy sources.^[^ ^1^ ^]^ Electrochemical conversion technologies, such as fuel cell powering,^[^ ^2^ ^]^ water electrolysis,^[^ ^3^ ^]^ electrochemically rechargeable metal–air batteries,^[^ ^4^ ^]^ chloralkaline process,^[^ ^5^ ^]^ and electrocatalytic nitrogen fixation^[^ ^6^ ^]^ are the promising strategies to achieve a sustainable energy future due to the advantages of using green energy to enable the catalytic conversion of abundant molecules (e.g., H_2_O, CO_2_, and N_2_) into value‐added products (e.g., H_2_, NH_3_, and hydrocarbons).^[^ ^7^ ^]^ To date, some electrocatalytic reactions have been widely studied, including hydrogen evolution reaction (HER),^[^ ^8^ ^]^ oxygen evolution reaction (OER),^[^ ^9^ ^]^ oxygen reduction reaction (ORR),^[^ ^10^ ^]^ chlorine evolution reaction(CER),^[^ ^11^ ^]^ and nitrogen reduction reaction (NRR).^[^ ^12^ ^]^ So far, many of these reactions rely on precious metals as the effective catalysts.

An industrially promising electrocatalyst must at least fulfil three requirements, including i) bulk‐ and liquid‐phase stability, ii) high activity and target‐product selectivity, and iii) low‐cost.^[^ ^13^ ^]^ Metal oxides (MOs) are a class of potentially stable catalysts that can be the alternatives to precious metals, especially under alkaline electrocatalytic conditions.^[^ ^14^ ^]^ Compared to precious metals, their much lower cost makes it possible to achieve commercial applications.^[^ ^15^ ^]^ Unfortunately, looking for stable, active, and target‐product selective MOs was generally based on an trial‐and‐error process of either experimental or first principle computational screening,^[^ ^13^ ^]^ which has consumed significant time, expenses, and human resources. Data mining from the open‐source computational databases can contribute to the search of unexplored promising materials (e.g., thermoelectric materials,^[^ ^16^ ^]^ optoelectronic semiconductors,^[^ ^17^ ^]^ and batteries^[^ ^18^ ^]^), which could also be a way‐out to effectively search promising catalysts.^[^ ^19^, ^20^ ^]^ The Materials Project database,^[^ ^21^ ^]^ the largest computational bulk material database reported to date, has included various physical properties of >140 000 materials, among which the thermodynamic phase diagrams^[^ ^22^, ^23^, ^24^ ^]^ and bulk Pourbaix phase diagrams^[^ ^25^, ^26^, ^27^ ^]^ can help assess the thermodynamic and aqueous stability of potential electrocatalysts.

To avoid catalyst aging and deactivation and guarantee a long‐term stability,^[^ ^28^ ^]^ an electrocatalyst first needs to have a bulk stability. The defined thermodynamically stable compounds possess an energy above hull (*E*_hull_) of zero and the lowest formation energy (*E*_Form_) in the thermodynamic phase diagram,^[^ ^29^ ^]^ while the aqueous stability for different electrochemical reactions can be confirmed by evaluating the Gibbs free energy difference (Δ*G*_pbx_) under aqueous conditions and the stable phase species that are mapped at the Pourbaix diagram.^[^ ^26^ ^]^ Bulk Pourbaix diagrams have been broadly used to assess the stability domains of materials depending on different potential and pH values in aqueous environments.^[^ ^27^ ^]^

Recently, data mining from Pourbaix diagrams in the Materials Project database has aided in determining 68 promising stable MO electrocatalysts under acidic conditions at ORR and OER potentials.^[^ ^30^ ^]^ However, different electrochemical reactions happen on distinct potentials, thus it is meaningful to design a searching engine based on the Pourbaix diagram dataset that can be extracted from the Materials Project database, in which researchers can quickly determine all potentially stable MOs by defining the certain pH and potential conditions. Moreover, considering the electrochemical surface states of MOs under operating conditions is essential because a surface can be pre‐covered by the adsorbates (e.g., HO*, O*, and H*) generated by the electrochemistry‐driven water activation,^[^ ^31^, ^32^, ^33^ ^]^ leading to deviations from their pristine surface, ultimately impacting the stability and activity of MOs.^[^ ^34^, ^35^ ^]^ The phenomenon of surface coverage has been observed in dual‐atom catalysts^[^ ^32^ ^]^ and various X‐ides (e.g., oxides, nitrides, carbides, and hydroxides) catalysts.^[^ ^31^, ^34^ ^]^ Additionally, the pH of the surrounding environment significantly influences the catalyst's performance. Therefore, a comprehensive analysis considering both the electrochemical surface states and pH effects is indispensable to unravel the intricate origin of MOs' catalytic activity.^[^ ^15^ ^]^ A notable example to illustrate the necessity of electrochemical surface state and pH effect analyses can be found in our recent work:^[^ ^34^ ^]^ only when considering both these two effects, can we fully understand the superior performance of ZrN in alkaline ORR.

Motivated by the current stages, we analyze the essential opportunities and challenges in identifying promising electrocatalysts initialized by data mining via a highly integrated analytical strategy. Our approach combined data mining, experimental verification, electrochemical surface state analyses, and pH‐field coupled microkinetic modeling to make the prediction results more accurate and reliable. This approach can help avoid the uncertainty associated with density functional theory (DFT) calculations.^[^ ^36^, ^37^ ^]^ By reporting an observed discrepancy between computational data mining and experimental observations when attempting to identify low‐cost MO electrocatalysts, the key challenges of data mining are discussed. First, we built a bulk Pourbaix diagram dataset under specific pH and potentials for 1159 bulk‐stable MOs that show the potential thermodynamic stability. A searching engine was designed to define stable MOs under different conditions based on the Pourbaix diagram dataset by setting two criteria: i) values of ΔGpbx < 0.5 eV atom^−1^ and ii) the existence of solid‐phase domains. Thereafter, Sb‐ and W‐based MOs generally fulfill the stability criteria of the Pourbaix diagram dataset under some electrochemical conditions especially acidic ORR. Based on the aqueous stability diagram, Sb_2_WO_6_ is stable under ORR conditions in acidic media but rather unstable under high‐pH ORR conditions. However, this contradicts to our experimental verification that Sb_2_WO_6_ has high stability under both acidic and alkaline ORR conditions, while it even displayed an outstanding four‐electron (4*e*) ORR activity under alkaline conditions. Based on the subsequent surface state analyses and pH‐field coupled microkinetic modeling, we found that the Sb_2_WO_6_ surface will undergo an electrochemical passivation that leads to a highly stable and 4*e*‐ORR active surface. These highly interactive analyses suggest that though data mining is promising to explore electrocatalysts, a refined strategy needs to be further developed by considering the electrochemistry‐induced surface stability and activity.

## 2 Results and Discussion

### 2.1 Data Mining for Aqueous Stable MOs

**Figure 1:**
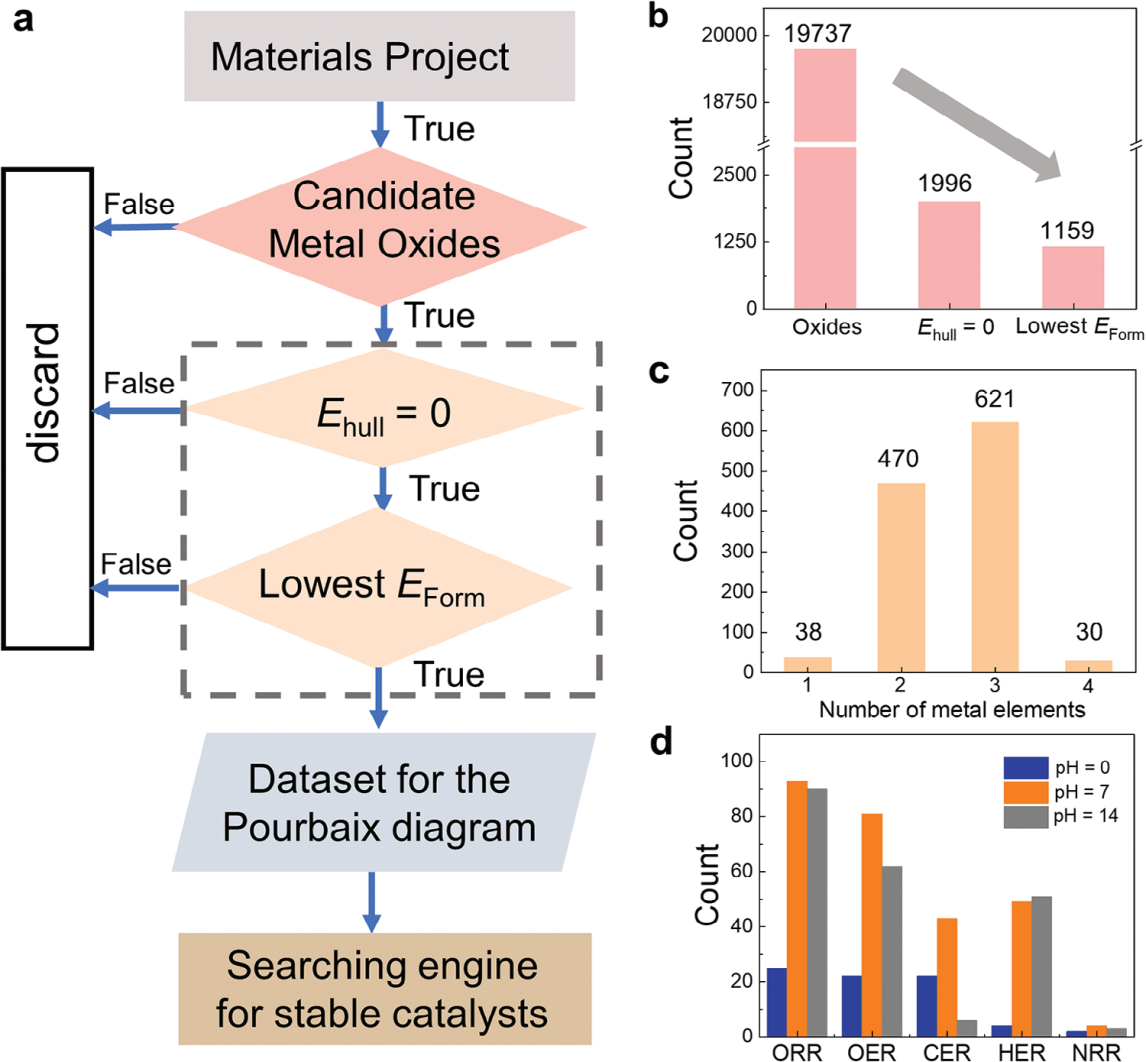
Workflow of the data mining process to identify aqueous stable MOs. a) Flow chart of the data mining process for identifying stable MOs from the Materials Project database. b) The number of MOs after each screening step and c) bulk‐stable MOs sorted by the number of metal elements. d) The number of aqueous stable MOs under different conditions.

To identify potential MO electrocatalysts, we performed data mining for aqueous stable MO electrocatalysts from the Materials Project (Database version: V2021.03.22) by the following procedures, as shown in **Figure** **1**a.

First, we identified cost‐effective MO structures from the database by discarding any compounds that contain noble, radioactive, halogen, gaseous, and organic elements. Some materials with elements (e.g., some lanthanides) that cannot be precisely described by available functional methods of the database were also temporarily excluded.^[^ ^38^ ^]^ Next, we selected MOs with *E*_hull_ = 0 eV, and then selected the MOs with the lowest *E*_Form_ based on the thermodynamic stability theory to ensure material's synthesizability.^[^ ^39^ ^]^

Following this procedure, we identified 1159 MOs with bulk‐phase stability from the total of 19737 MOs in the database (Figure 1b), including 38 unitary, 470 binary, 621 ternary, and 30 quaternary compounds (Figure 1c). The number of various elements present in our dataset is shown in Figure S1 (Supporting Information). The top ten elements with the highest occurrence are Ba, Li, Ge, K, V, Na, Sr, Sb, Mn, and Ca. Subsequently, their bulk Pourbaix diagram dataset, which contains the Δ*G*_pbx_ and stable phase species within pH between 0 to 14 and a potential window of −1.2 to 2.16 V referred to standard hydrogen electrode (SHE) (V_SHE_) was created. Aqueous stable materials under different pH and potentials should, in principle, have a Δ*G*_pbx_ <0.5 eV atom^−1[^ ^26^ ^]^ and remain in solid‐phase. Δ*G*_pbx_ is defined as the Gibbs free energy of decomposition of a material into Pourbaix stable domains at specific pH and potential conditions. K. A. Persson and co‐workers^[^ ^26^, ^30^ ^]^ found that materials with predicted Δ*G*_pbx_ <0.5 eV atom^−1^ can potentially remain stable in aqueous environments, which is attributed to self‐passivation and formation of more stable solid‐state surface phases. After confirming the cutoff of Δ*G*_pbx_ values, we should consider the existence of solid phase species in the bulk Pourbaix diagram to ensure the stability of this material. Following these criteria, we developed an online search engine with user‐interface (https://doi.org/10.50974/00137200) to assist researchers quickly access stable MOs at a customized pH and potential combination based on this dataset.

Given that the catalysts are often cycled over a variety of operating potentials at a certain pH in realistic experiments,^[^ ^30^, ^40^ ^]^ the aqueous stability of these materials was further screened in a definite range of potentials, i.e., 0.6 to 1.0 V_SHE_ for ORR, 1.23 to 2.0 V_SHE_ for OER, −0.8 to 0 V_SHE_ for HER, 1.36 to 2.16 V_SHE_ for CER, and −1.2 to 0 V_SHE_ for NRR, with pH 0. These potential windows are decreased by 0.059 eV per pH on the SHE scale, except for CER, which is pH‐independent.^[^ ^41^ ^]^ We applied the screening standards, i.e., that the maximum Δ*G*_pbx_ is <0.5 eV atom^−1^ and the existence of solid domains in a certain range of potentials, and those MOs, which are stable at different conditions and potentials relevant to ORR, OER, HER, CER, and NRR were determined. Figures S2–S4 (Supporting Information) show the typical examples of the interface of our search engine for identifying stable MOs for ORR, OER, and HER, exemplifying the stable MOs across a pH range from 0 to 14. The number of aqueous stable MOs under different conditions are shown in Figure 1d. Under acidic conditions (pH = 0, blue bars in Figure 1d), 25, 22, and 22 MOs are found to be stable for ORR, OER, and CER, respectively, while only 4 and 2 MOs are respectively stable for HER and NRR. For an alkaline environment (pH 14, grey bars in Figure 1d), much more MOs can survive for ORR (90), OER (62), and HER (51), but less MOs can survive for CER (6). As for neutral conditions, there are almost always more stable materials compared to acidic and alkaline conditions.

### 2.2 Statistical Analysis for Aqueous Stable MOs

**Figure 2:**
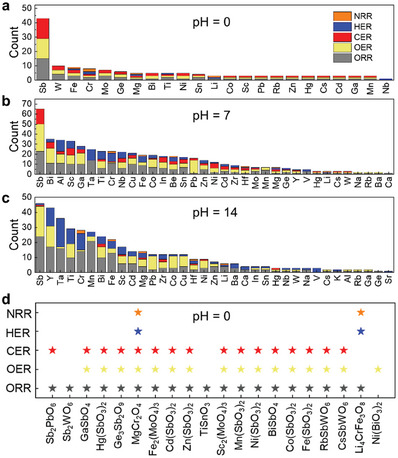
Statistical analysis of the defined stable catalysts after data mining. Statistics of the metal elements from the defined stable MOs at a) pH 0, b) pH 7, and c) pH 14 for NRR, HER, CER, OER, and ORR. d) Identified stable MOs under pH 0 for the five electrochemical reactions. Results under other pH conditions are shown in Figure S5 (Supporting Information).

**Figure** **2** shows the statistical analyses of the metal elements from the defined stable MOs under different pH and reactions. Interestingly, Sb‐based MOs always have the highest number among all the stable materials for ORR, OER, and CER under acidic and neutral environments, and for ORR and OER under alkaline environments (Figure 2a–c), and these identified stable Sb‐based MOs for different reactions are shown in Figure S5 (Supporting Information). Cr for HER and NRR at pH 0 (Figure 2a), Al/Ta and Cr for HER and NRR at pH = 7 (Figure 2b), Bi, Ta, and Cr for CER, HER, and NRR at pH 14 (Figure 2c) appear the most counts compared to other elements. We normalized the number of each element by dividing it by the corresponding element count in our dataset, as shown in Tables S1–S3 (Supporting Information). Among the 110 Sb‐containing compounds in our dataset, 13.6%, 12.7%, and 12.7% of them are stable at pH 0 for ORR, OER, and CER, respectively (Tables S1, Supporting Information). This still constitutes a great proportion compared to other elements, *e*.*g*., only 4.9% (ORR), 3.7% (OER), and 3.7% (CER) of W‐based compounds can survive among the 81 W‐containing compounds. At pH 7 (Table S2, Supporting Information) and pH 14 (Table S3, Supporting Information), the proportion of Sb‐based compounds increases, suggesting that Sb‐based materials always have a greater ability to withstand extreme conditions like acidic conditions compared to other materials. Note that under low‐potential conditions (e.g., HER and NRR), some less stable MOs may form oxygen vacancies that lead to an aqueous stable surface, which will be discussed later in this paper. Details of all potentially stable MOs at different conditions can be found in our search engine (https://doi.org/10.50974/00137200) by defining the pH and potential range.

Since unitary oxides (*i.e*., the MOs with only one metal element) have been extensively studied,^[^ ^42^ ^]^ herein, we mainly focus on analyzing binary and ternary MOs, i.e., A*_x_* B*_y_* O*_z_*/A*_x_* B*_y_* C*_m_* O*_z_*, where A, B, and C are different metal elements and *x*, *y*, *m*, and *z* are the stoichiometric numbers. Figure 2d shows the binary and ternary MOs identified to be stable under pH 0 for different reactions. Interestingly, we found that (Hg/Cd/Zn/Mn/Ni/Co/Fe)(SbO_3_)_2_, (Sc/Fe)_2_(MoO_4_)_3_, (Ga/Bi)SbO_4_, (Cs/Rb)SbWO_6_, and Ge_3_Sb_2_O_9_ are generally stable at moderate and high potential reactions (i.e., ORR, OER, and CER). According to the results, MgCr_2_O_4_ is the only material that is stable for all five electrocatalysis, while Sb_2_WO_6_ and TiSnO_3_ can only survive for ORR. Except for Sb_2_WO_6_, MgCr_2_O_4_, TiSnO_3_, RbSbWO_6_, and Li_4_CrFe_3_O_8_, other MOs were ever predicted as stable candidates for ORR in previous works.^[^ ^30^ ^]^ Because Sb and W display more frequently under acidic ORR conditions, we selected Sb_2_WO_6_ (the bulk Pourbaix diagram is shown in Figure S6, Supporting Information) for the subsequent experimental investigations.

### 2.3 Experimental Validation

**Figure 3:**
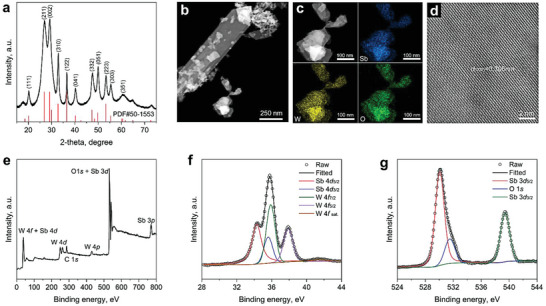
Structure characterization of the as‐synthesized Sb_2_WO_6_ catalyst. a) XRD pattern of the as‐prepared catalyst, showing good agreement to Sb_2_WO_6_ in an orthorhombic structure (PDF#50‐1553). b) A dark field STEM image, c) EDX mapping results, and d) a high‐resolution HAADF‐STEM image of Sb_2_WO_6_. e) XPS survey scan and high‐resolution spectra of f) Sb 4*d*, W 4*f*, and g) O 1*s*, and Sb 3*d* regions.

We synthesized Sb_2_WO_6_ by a hydrothermal method (see details in the Experimental Section). Scanning electron microscope (SEM) characterizations showed that the as‐synthesized Sb_2_WO_6_ are nanoplates with an average thickness of ≈20 nm (Figure S7, Supporting Information). Inductively coupled plasma optical emission spectroscopy (ICP‐OES) measurement afforded an Sb to W molar ratio of 1.99 ± 0.01, close to the theoretical stoichiometric value of 2:1. Its X‐ray diffraction (XRD; **Figure** **3**a) is in good agreement with standard Sb_2_WO_6_ in an orthorhombic structure (PDF#50‐1553). The 2D‐nanoplate morphology of the as‐synthesized Sb_2_WO_6_ can be clearly observed under a high‐angle annular dark field scanning transmission electron microscope (HAADF‐STEM; Figure 3b). A uniform distribution of Sb, W, and O elements can be found from the corresponding energy‐dispersive X‐ray (EDX) spectroscopy elemental mapping results, displayed in Figure 3c. EDX elemental analysis also offered an atomic ratio of 2.00: 0.99: 6.04, showing good agreement with the ICP‐OES measurement. Clear lattice fringes with a spacing of 0.306 nm can be found from the atomic resolution STEM image displayed in Figure 3d, corresponding to the (002) plane of the orthorhombic Sb_2_WO_6_.

X‐ray photoelectron spectra (XPS) of the Sb_2_WO_6_ catalyst were also collected. The spectroscopic features corresponding to the Sb, W, and O elements can be assigned in the survey scan displayed in Figure 3e accordingly. No residual Na or Cl element can be found. The high‐resolution XPS spectra of the Sb 3*d*/4*d*, W 4*f*, and O 1*s* features are shown in Figure 3f–g, respectively. Spectra deconvolution revealed the existence of Sb^3+^, W^6+^, and O^2−^ in the form of MOs (detailed peak positions and assignments are tabulated in Table S4, Supporting Information). These characterization results confirmed the successful synthesis of Sb_2_WO_6_.

**Figure 4:**
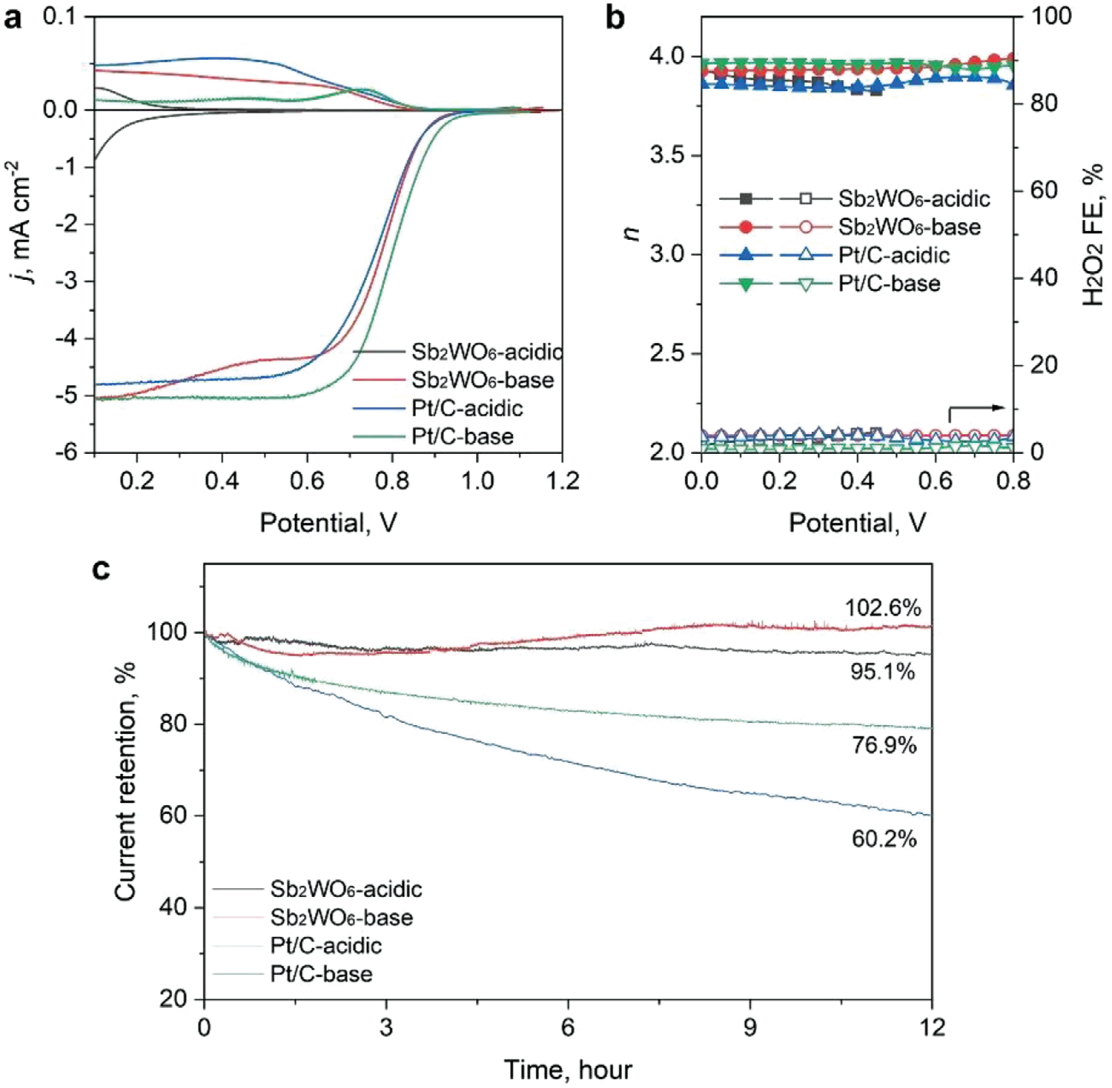
ORR activity and stability in acidic and alkaline electrolytes. a) ORR LSV curves, and b) the calculated electron transfer number (*n*) and H_2_O_2_ Faradaic efficiency obtained in acidic and alkaline electrolytes. c) Chronopotentiometry curves of the catalyst in different electrolytes collected for 12 h.

The ORR performance of the Sb_2_WO_6_ catalyst was then measured in an acidic 0.1 M HClO_4_ (pH = 1.3) and an alkaline 0.1 M KOH (pH = 12.6) electrolytes. The linear sweep voltammetry (LSV) curves obtained on a rotary ring‐disk electrode (RRDE) at 1600 rpm are displayed in **Figure** **4**a. In the alkaline electrolyte, Sb_2_WO_6_ catalyst exhibits an onset potential of 0.94 V versus a reversible hydrogen electrode (V_RHE_) to reach a disk current density of 0.05 mA cm^−2^ and half‐wave potential (*E*_1/2_) of 0.78 V_RHE_, which are close to that of a commercially available 20 wt.% Pt/C catalyst (0.98 and 0.80 V_RHE_, respectively). It shows a reduced ORR performance in the acidic electrolyte, which is in good agreement with recent reports that MOs are intrinsically less active under acidic conditions.^[^ ^15^ ^]^ However, Sb_2_WO_6_ can well‐maintain a four‐electron transferred ORR pathway in both electrolytes, as shown in Figure 4b. We further loaded the catalyst on a carbon cloth electrode and performed chronoamperometric tests under both acidic and alkaline conditions (Figure 4c). It can be found that the Sb_2_WO_6_ exhibits a superior stability performance to that of the Pt/C in both acidic and basic electrolytes. After biasing at 0.2 V_RHE_ in the acidic electrolyte, the Sb_2_WO_6_ can still retain ≈95.1% of its initial current, superior to that of 60.2% of Pt/C when biased at a similar potential after 12 h. Chronoamperometric tests were also performed in alkaline electrolytes at 0.6 V_RHE_. Interestingly, we found that after a slight current loss for the initial 2 h when biased at 0.6 V_RHE_, the activity of Sb_2_WO_6_ slightly increased to ≈102.6% after the 12‐h test, which is better compared to the 76.9% current retention for Pt/C. We also conducted the experiment to show the ORR performance of blank carbon black (CB) substrate as shown in Figure S8 (Supporting Information). The CB substrate shows inferior ORR activity and a much higher H_2_O_2_ selectivity. Therefore, the 4e‐ORR performance found on the Sb_2_WO_6_ sample can be attributed to the metal oxide only. Therefore, it is intriguing to elucidate the mechanisms of the improved performance in alkaline electrolytes.

### 2.4 Experimental and Theoretical Understanding of the ORR Stability

**Figure 5:**
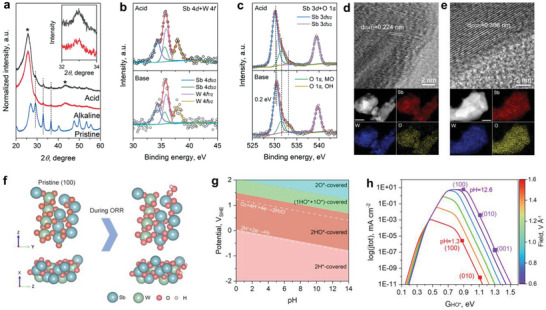
Post‐stability test characterization and theoretical insights. a) XRD patterns. Inset shows a magnified region of the (310) peak. High‐resolution XPS spectra in b) Sb 4*d* + W 4*f* and c) Sb 3*d* + O 1*s* regions. HR‐TEM image and EDX elemental mapping results of post‐test catalyst in d) acidic and e) alkaline electrolytes. The scale bar for EDX mapping results is 50 nm. f) The stoichiometric pristine surface of Sb_2_WO_6_ (100) and its identified electrochemistry‐induced surface state under ORR conditions from different views. g) Calculated surface Pourbaix diagram of Sb_2_WO_6_. h) pH‐dependent microkinetic modeling of the ORR process at 0.8 V_RHE_.

We performed comprehensive structural tests over the post‐stability test catalysts and combined with additional DFT calculations to better understand our observation. XRD patterns of the post‐test catalysts were collected and displayed in **Figure** **5**a in comparison to that of the pristine catalyst. Despite the strong peaks of the carbon cloth substrate (marked by * at ≈25° and 42°), Sb_2_WO_6_ features can still be identified. We find that the full width at half maximum (FWHM) of the Sb_2_WO_6_ (002), (310), and (122) peaks (marked by dashed lines) collected from the alkaline electrolyte tested catalysts are all ≈20–30% wider to that obtained from the acidic electrolyte (inset of Figure 5a), indicating its stronger crystal structure distortion.

This observation is also confirmed by Raman spectra (Figure S9a, Supporting Information) collected on these samples. The spectrum of the pristine Sb_2_WO_6_ is in good agreement with previous reports.^[^ ^43^ ^]^ Deconvolution of the broad peak at 874 cm^−1^ using two Lorentz peaks offered a peak area ratio of 0.89 ± 0.04, indicating its good crystallinity (Figure S9b, Supporting Information). While major Raman features can be largely retained after the stability test, this peak area ratio has dropped to 0.84 ± 0.03 and 0.69 ± 0.04 for acidic and alkaline tested catalysts, respectively. Compared to the reported values, these intensity ratios suggest that the catalyst bulk crystallinity can be largely retained,^[^ ^43^ ^]^ but the substantially reduced value for the alkaline tested sample implies possible structural transformation during ORR. This observation is further supported by the measured electrochemical active surface area (ECSA) of these samples. After testing in the alkaline electrolyte, the ECSA has slightly increased by ≈2.2% (Figure S10, Supporting Information). In contrast, the ECSA of the catalyst tested in acid is comparable to the as‐synthesized catalyst.

We further probed the surface chemical information of the post‐stability test catalysts by XPS. Compared to that of the as‐synthesized catalyst (Figure 3f), negligible changes can be found in the W 4*f* + Sb 4*d* region (Figure 5b), except for a small blueshift of ≈0.1 eV found for Sb 4*d* peaks after testing in alkaline electrolyte. A more obvious blueshift of ≈0.2 eV is found for the Sb 3*d* feature after testing in an alkaline electrolyte (Figure 5c), suggesting the formation of high valence Sb^3+δ^ species due to the surface structure transformation. Meanwhile, the O 1*s* peak analysis reveals the formation of M‐OH species (≈533.1 eV) in the post‐test catalysts. Based on the peak area, the M‐OH feature takes ≈38% of surface oxygen species for the alkaline tested sample, which is much greater than that of the acidic case (11%). The XRD, Raman, and XPS spectroscopic results suggest that under alkaline conditions, the Sb_2_WO_6_ surface may adsorb hydroxides and form a low crystalline layer, and this speculation is further confirmed by high‐resolution transmission electron microscopy (HR‐TEM) characterization results displayed in Figure 5d,e.

As shown in Figure 5d, the lattice of the catalyst tested in acid shows minimum structure alternation, with the (041) plane extending from the bulk to the surface. In contrast, after testing in alkaline conditions, a surface with less ordered structure can be found, as shown in Figure 5e. This surface layer with reduced crystallinity could result in the corresponding features observed in various spectroscopic characterizations discussed above. Besides, EDX elemental mapping results suggest that Sb, W, and O elements are still uniformly distributed in the post‐test catalysts and revealed an Sb to W atomic ratio of 2.00 ± 0.02 for the catalyst after testing in acid, which is identical to that of the as‐synthesized catalyst. However, a slightly increased Sb to W atomic ratio of 2.03 ± 0.02 is found for the catalyst tested under alkaline conditions. Indeed, a trace amount of W was found from the alkaline but not the acidic electrolyte after testing. While Pourbaix plots and experiments have suggested that tungstate is stable in acid,^[^ ^44^, ^45^ ^]^ we could conclude that under alkaline conditions, the surface W species, at its highest valence state (+6), may dissolute and the exposed Sb–O layer. The Sb–O surface is then passivated with OH, forming a slightly amorphous layer and protecting the inner crystals from further degradation. This is also in good agreement with the XPS measurements that only Sb exhibits a slightly shifted binding energy.

To further understand the experimental observations discussed above, we further provide theoretical insights into the electrochemistry‐induced surface states and ORR activities of Sb_2_WO_6_ under acidic and alkaline conditions (computational details can be found in the Experimental Section). The (100) surface of Sb_2_WO_6_ was selected for the theoretical analysis by considering its lowest surface energy (detailed discussion can be found in Supplementary Information, Figure 5f; Figures S11 and S12, and Table S5, Supporting Information). Notably, this surface is orthogonal to the (002) and (041) lattice fringes identified in the HR‐TEM images of the pristine and post‐stability test catalysts, respectively (Figure 5d,e). Considering the orthorhombic crystalline structure and the 2D nanosheet morphology of the Sb_2_WO_6_ catalyst (Figure 3c), it is safe for us to assign the dominating exposed surface to the (100) plane. The surface Pourbaix diagram^[^ ^46^ ^]^ was calculated (Figure S11, Supporting Information), and we plotted the most stable surface as the function of pH and potential against a SHE (V_SHE_) to probe the surface state information of Sb_2_WO_6_. Figure 5g presents various electrochemical surface states, aligning with findings that at moderate or high potentials (e.g., V_SHE_ > 0 V at pH 0), the surfaces tend to be covered by O‐containing molecules, while surfaces with vacancies or hydrogen coverage may form at low potentials (e.g., V_SHE_ < 0 V at pH 0).^[^ ^31^ ^]^ In the ORR potential window, i.e., between the dash lines marking for ORR and HER, the catalyst surface is occupied by up to 1/2 monolayer (ML) HO*. This suggests an electrochemical oxidation process of Sb_2_WO_6_ and the blocking of some surface cation sites under ORR potentials, which can also result in a semi‐passivation layer that prevents the cations from leaching out. This is also in good agreement with the strong HO* features detected by XPS on the Sb_2_WO_6_ surface after the ORR stability test under alkaline conditions (Figure 5b), which could contribute to the origin of the unexpectedly good alkaline ORR stability. Similar surface‐stabilization mechanisms were also identified on some recent research under oxidizing condition reactions, such as Mn‐oxides for OER^[^ ^47^ ^]^ and La‐oxides for ORR.^[^ ^48^ ^]^ Therefore, Sb_2_WO_6_ possesses high stability and activity under alkaline conditions even though the bulk Pourbaix information based on data mining suggests the opposite stability information. Consequently, it is crucial to conduct a comprehensive analysis that takes into account the surface's electrochemical stability and activity after performing data mining.

This electrochemical surface state analysis also suggests that the subsequent electrocatalytic activity analysis should be performed over a more realistic surface coverage instead of a pristine stoichiometric Sb_2_WO_6_. Therefore, a pH‐field coupled microkinetic modeling was performed with the consideration of pH‐ and field‐dependent energetics of all possible elementary steps.^[^ ^15^, ^49^ ^]^ Relevant details can be found in the Supporting Information. We also plotted the predicted activities of surfaces (100), (001), and (101), as shown in Figure 5h. The ORR activity model follows a “volcano‐shape” as a function of HO* binding free energy, which is consistent with the Sabatier principle for heterogeneous catalysis.^[^ ^50^ ^]^ Interestingly, the ORR activity (*i.e*., the simulated current density at 0.8 V_RHE_) of Sb_2_WO_6_ (100) is higher than those of other surfaces, and the ORR activity under alkaline conditions of (100) (purple point, pH 12.6) can approach the peak of the volcano, which is significantly higher than that under acidic conditions (red point, pH = 1.30), in excellent agreement with experimental observations in Figure 4. These results also suggest that the Sb_2_WO_6_ surface transformation under alkaline conditions not only enhances the stability of the catalyst but also endows the catalysts with an outstanding ORR activity.

The above‐combined analyses, which crosses data mining, electrochemical surface state analysis, advanced pH‐dependent microkinetic modeling, and experiments, clearly indicate that there is a possibility of dismissing potentially stable and active MOs if one only relies on the screening of bulk stability results from data mining. The lack of surface state (i.e., coverage) information can cause the overlooking of some electrochemical oxidation processes that may stabilize the surface under electrocatalytic conditions. Likewise, at low‐potential reactions (e.g., HER and NRR), there is also a possibility that some less‐stable MOs can form a metallic or semi‐metallic phase by oxygen vacancy formation that could be stable under the electrochemical potentials of interest.^[^ ^14^ ^]^ This essential surface information cannot be captured from the currently available computational database, and thus it would be easy to dismiss many valuable electrocatalysts if a catalyst search process only refers to the data mining results from the materials’ bulk stability. To aid in the predictive power of data mining for catalyst surfaces, a refined strategy that integrates the electrochemical surface information needs to be further developed by considering the electrochemistry‐induced surface stability and activity.

## 3 Conclusion

In this paper, we have analyzed the opportunities and challenges of data mining from computational materials database by reporting a discrepancy between computational data mining and experimental observations when attempting to identify low‐cost MO electrocatalysts. Based on a novel search engine capable of identifying stable MOs at the pH and potentials of interest, Sb_2_WO_6_ attracted our attention among the identified stable MOs in acid. Based on the aqueous stability diagram, Sb_2_WO_6_ is stable under ORR in acidic media but rather unstable under high‐pH ORR conditions. However, this contradicts to our subsequent experiments that Sb_2_WO_6_ has high stability under both acidic and alkaline ORR conditions, while it even displayed an outstanding 4*e*‐ORR activity under alkaline conditions. Based on the surface state analyses and pH‐field coupled microkinetic modeling, we found that the Sb_2_WO_6_ surface will undergo an electrochemical oxidation process under ORR potentials that lead to a highly stable and 4*e*‐ORR‐active surface. These highly interactive analyses conclude that though data mining is promising for exploring electrocatalysts, a refined strategy needs to be further developed by considering the electrochemistry‐induced surface stability and activity.

## 4 Experimental Section

### Data Mining

Python codes were developed based on the Python Materials Genomics (Pymatgen) library^[^ ^51^ ^]^ for the data mining of the Materials Project database. The “from pymatgen.ext.matproj import MPRester” can activate the Materials Project Applicant Programming Interface (API)^[^ ^52^ ^]^ that allows researchers to obtain materials’ information from the database. The data mining for stable MO catalysts was enabled by comparing phase diagrams and Pourbaix diagrams obtained from “pymatgen.analysis.phase_diagram” and “pymatgen.analysis.pourbaix_diagram”, respectively. An open‐source app framework, *Streamlit*, was employed to build the search engine of our results.

### DFT Calculations

All calculations were performed using DFT implemented in the Vienna Ab initio Simulation Package (VASP).^[^ ^55^, ^56^ ^]^ The generalized gradient approximation method with the revised Perdew–Burke–Ernzerhof (RPBE) functionals^[^ ^57^, ^58^ ^]^ and projector augmented wave (PAW)^[^ ^59^ ^]^ method were employed. The energy cutoff and force convergence criteria were set to 400 eV and 0.05 eV Å^−1^, respectively.^[^ ^60^ ^]^ A *k*‐mesh grid (3 × 3 × 1) of Monkhorst‐Pack scheme was applied for slab surfaces.^[^ ^61^ ^]^ For each slab model, a vacuum of 15 Å was set to the *z*‐direction to separate images. The zero‐point energy (ZPE) and entropic corrections are referred from previous studies, with the temperature set to 298.15 K.^[^ ^62^ ^]^ Solvation corrections were added to the HO* species due to the strong hydrogen bonding effects that further stabilize the bonding strengths, with the values referred from Ref.[63] For those HO* generated from the H* coverage, these solvation effects were also considered.

### Surface Pourbaix Diagrams

Surface Pourbaix diagrams were constructed to identify the stable states on the catalyst surface as a function of pH and the electrode potential (*V*). Assuming equilibrium between the adsorption sites and water, the total free energy (*G*) of the system is written as a function of the chemical potential of protons ($\mu_{H^{+}}$) and electrons ($\mu_{e^{-}}$) as:

(1) $$G\;=G_{bare}\;+mG_{H_{2}O}-G_{tot}-\left(2m-n\right)\left(\mu_{H^{+}}-\mu_{e^{-}}\right)$$

where *G_bare_*, $G_{H_{2}O}$, and *G_tot_* represent the free energies of the pristine surface, water molecule, and the surface with adsorbed species. By applying the computational hydrogen electrode (CHE) scheme,^[^ ^53^ ^]^ $\mu_{H^{+}}$ and $\mu_{e^{-}}$ are treated as one variable, $\mu_{H^{+}+e^{-}}$. For every proton–electron transfer step, the chemical potentials of the protons and electrons are given by the free energy of H_2_ (g) via H^+^ + e^−^ → 1/2H_2_ (g), straightforwardly computed by DFT.^[^ ^46^, ^54^ ^]^ $\mu_{H^{+}+e^{-}}$ is obtained by:

(2) $$\;\mu_{H^{+}+e^{-}}=\frac{1}{2}\;G_{H_{2}}^{0}-eV_{SHE}-2.303k_{B}T\cdot pH$$

where *e* is the elementary charge of electron, and $G_{H_{2}}^{0}$is the standard free energy of H_2_ (g). *V_SHE_* is the potential referred to standard hydrogen electrode (SHE), *k_B_* is the Boltzmann constant, and *T* is the temperature (set to 298.15 K).

### Experimental Methods

Sodium tungstate dihydrate (Na_2_WO_4_∙2H_2_O, ≥ 99%), antimony chloride (SbCl_3_, ≥ 99%), perchloric acid (HClO_4_, 70%), and potassium hydroxide (KOH, semiconductor grade, 99.99%) were purchased from Sigma–Aldrich and used without purification. Deionized water (DI H_2_O) was obtained from a Millipore water system with a resistance of 18.2 MΩ∙cm. Sb_2_WO_6_ was synthesized by hydrothermal reaction. About 137 mg SbCl_3_ (0.6 mmol) and 99 mg Na_2_WO_4_∙2H_2_O (0.3 mmol) were dissolved in 15 mL DI H_2_O separately by vigorous stirring. The tungstate solution was then added to the SbCl_3_ solution drop wisely under stirring. After further mixing for 1 h, the solution was transferred to a Teflon‐lined autoclave (Parr, 45 mL). The reactor was kept at 180 °C for 12 h before the solids were recovered by centrifugation and washed with excessive DI H_2_O before dried at 70 °C overnight.

XRD pattern of the samples were collected on a PANalytical X'Pert Powder diffractometer using a Cu Kα source (*λ* = 1.5406 Å) at a scan rate of 2 degree per minute. XPS measurement was performed on a Thermo Scientific K‐Alpha+ spectrometer with an Al‐Kα source (1486.3 eV). The elemental composition was further confirmed by ICP‐OES on a Perkin Elmer Avio 500 spectrometer. SEM images were collected on a Zeiss Ultraplus microscope. Spherical aberration‐corrected HAADF‐STEM images were obtained on an FEI Themis‐Z microscope. After electrochemical test, the catalyst was recovered, and its surface chemical composition and atomic structure was characterized by XPS and HR‐TEM.

The ORR performance was evaluated under a three‐electrode configuration on an CHI760 electrochemical workstation in acidic 0.1 m HClO_4_ (pH 1.3) and alkaline 0.1 m KOH (pH 12.6) electrolytes. A rotary ring‐disk electrode (RRDE, Pine, E6R2, calibrated collection efficiency *N* = 0.379) with a glassy carbon disk (GCE, 0.2375 cm^2^) and a Pt ring (0.2355 cm^2^) was used as the working electrode. A pre‐calibrated Ag/AgCl electrode (3 m KCl filling, Basi, MF‐2056) and a saturated calomel electrode (SCE) were used as the reference electrode for acidic and alkaline electrolytes, respectively. A graphite electrode (Pine, AFCTR3B) was used as the counter electrode. All reported potentials were calibrated to versus a reversible hydrogen electrode (RHE). The catalyst ink was prepared by dispersing 5 mg of the as‐synthesized catalyst in 1 mL water/isopropanol solution (1/9 = v/v, containing 0.05% Nafion 117) with 1 mg carbon black (Vulcan XC‐72R) by 30 min bath sonication. The ink was drop casted on the disk electrode at a Sb_2_WO_6_ loading of 0.2 mg cm^−2^. LSV polarization curves were collected in O_2_ saturated electrolytes at a scan rate of 5 mV s^−1^ without *i*R‐compensation at a rotation of 1600 rpm. The ring electrode was biased at 1.2 V_RHE_. The currents were background‐corrected with the currents obtained in Ar‐saturated electrolytes. The electron transfer number (*n*) and the corresponding H_2_O_2_ Faradaic efficiency (FE_H2O2_, %) were calculated using the following equation:

(3) $$n\;=\;4\times \frac{i_{disk}}{i_{disk}+\frac{i_{ring}}{N}}$$

(4) $$\;FE_{H2O2}=\;100\%\times \frac{\frac{i_{ring}}{N}}{i_{disk}}$$

To perform the chronoamperometric test, another catalyst ink was prepared by dispersing the as‐prepared Sb_2_WO_6_ catalyst in a 1/9 H_2_O/IPA solution at 10 mg mL^−1^ without addition of carbon black and ionomers. The catalyst was loaded on a carbon cloth gas‐diffusion layer electrode at 1 mg cm^−2^. The chronoamperometric test was performed in three‐electrode configuration and the catalyst was biased at either 0.2 V_RHE_ (in 0.1 m HClO_4_) or 0.6 V_RHE_ (in 0.1 m KOH) for 12 h and the currents were recorded. After the test, the solids were recovered for characterization.

## Conflict of Interest

The authors declare no conflict of interest.

## Supporting information

Supporting Information

## Data Availability

The data that support the findings of this study are available from the corresponding author upon reasonable request.
