# Supplementary material for: Identifying Stable Electrocatalysts Initialized by Data Mining: Sb2WO6 for Oxygen Reduction
Source: Adv Sci (Weinh). 2023 Dec 7;11(5):2305630. doi: 10.1002/advs.202305630 (PMC10837344; doi:10.1002/advs.202305630)
Supplement: Supplementary file 1 — Supporting Information [file ADVS-11-2305630-s001.pdf]

ADVANCED SCIENCE
Open Access

Supporting Information

for *Adv. Sci.*, DOI 10.1002/advs.202305630

Identifying Stable Electrocatalysts Initialized by Data Mining: Sb_2WO_6 for Oxygen Reduction

Xue Jia, Zixun Yu, Fangzhou Liu, Heng Liu, Di Zhang, Egon Campos dos Santos, Hao Zheng, Yusuke Hashimoto, Yuan Chen, Li Wei and Hao Li**

Supporting Information for

Identifying Stable Electrocatalysts Initialized by Data Mining: Sb_2WO_6 for Oxygen Reduction

Xue Jia[1,#], Zixun Yu[1,2,#], Fangzhou Liu [2], Heng Liu[1], Di Zhang[1,3], Egon Campos dos Santos[1], Hao Zheng[1], Yusuke Hashimoto[4], Yuan Chen[2], Li Wei[2,*], and Hao Li[1,*]

[1] Advanced Institute for Materials Research (WPI-AIMR), Tohoku University, Sendai 980-8577, Japan

[2] School of Chemical and Biomolecule Engineering, The University of Sydney, Darlington 2006, NSW, Australia

[3] State Key Laboratory of Mechanical System and Vibration, Shanghai Jiao Tong University, Shanghai 200240, P.R. China

[4] Tohoku Forum for Creativity, Tohoku University, Sendai 980-8577, Japan

[#] Equally contribution

* Corresponding author: E-mail: l.wei@sydney.edu.au (W. L.), li.hao.b8@tohoku.ac.jp (H. L.)

1. How to define the stable materials based on phase species

After confirming that ΔG_{pbx} is lower than 0.5 eV/atom, we should consider the existence of solid phase species in the bulk Pourbaix diagram to ensure the stability of this material. Based on previous studies [1], we summarized the standard how to define the stable materials based on phase species: i) in a defined pH and potential range, if the phase species aligns with the composition of the compound or encompasses it, this compound is deemed to exhibit stability; ii) in a defined pH and potential range, if the phase species does not encompass the compound but nevertheless represent the solid phases across the entire potential range or within a broad potential range, this compound also can be considered to possess potential stability.

2. Details of pH-dependent microkinetic modeling methods

We used the Quantum Espresso code [2] to calculate the electric field effects. Electric fields were applied using a saw-tooth potential that corresponds to fields ranging from −0.8 to 0.8 V/Å. At each applied field, adsorbates were allowed to relax with a force convergence threshold of 0.05 eV/Å. We used the lowest energy conformation to predict the adsorbate energy under that field.

To describe the potential and pH dependence, we related the electric fields to both the standard hydrogen electrode (SHE) and reversible hydrogen electrode (RHE) potential using a parallel-plate capacitor model. The model is described by Equation (1):

$$\vec{E} = \frac{\sigma}{\varepsilon\varepsilon_0} = \frac{C_H(V_{SHE} - V_{PZC})}{\varepsilon\varepsilon_0} \tag{1}$$

where σ refers to charge density, ε_0 refers to the vacuum permittivity (8.85×10^{-12} F m^{-1}), ε refers to the dielectric constant (unitless), C_H refers to the Helmholtz capacitance (μF cm^{-2}), V_{SHE} refers to the potential *vs*. SHE, and V_{PZC} refers to the potential at the point of zero charges (PZCs) *vs*. SHE.

Fumagalli *et al.*[3] demonstrated that the dielectric constant of water near a surface is 2. The C_H can vary with the surface and potential but typically ranges between 20 and 30 over the majority of the potential range, with more elevated values near the PZC. For simplicity, we assumed a constant C_H of 25 μF cm^{-2} across all surfaces [4].

To measure an adsorbate's response to the field, we fitted a second-order polynomial to the calculations for each adsorbate across the range of fields. We then used Equation (2) to determine the values of the intrinsic dipole moment (μ) and polarizability (α).

$$G_{ads} = G_{ads}^{PZC} + \mu\vec{E} - \frac{\alpha}{2}\vec{E}^2 \qquad (2)$$

where $G_{\text{ads}}^{\text{PZC}}$ refers to the binding energy of adsorbate at the PZC, which corresponds to the energy calculated with no applied field. These fits, along with values for μ and α for each adsorbate on each electrode, are displayed in **Table S6**.

Our method for calculating binding energy dependence on the SHE potential differs from the methods employed by Ref.[5]. Our method requires the input of fixed PZC and C_H, whereas their method allows PZC and C_H to vary with adsorption but relies on the ability of implicit solvent methods to accurately predict these values. However, the accuracy of these previous predictions has been

called into question by several studies [6].

In this study, we utilized the implicit method VASPsol to determine the computational PZCs for Sb_2WO_6. We set the VASPsol parameters to default values, including a bulk dielectric constant $\varepsilon_k = 78.4$, width of dielectric cavity $\sigma = 0.6$, cutoff charge density $\rho_{cut} = 0.0025$ Å^{-3}, and a surface tension parameter of 0.525 meV/Å^2.

The C_{HE} was used to correct the binding energies for RHE dependence using Equation (3) as follows:

$$G_{ads} = G_{ads,V_{RHE=0}} - neV_{RHE} \quad (3)$$

where n refers to the number of electrons (relative to water), e refers to the charge of an electron, and V_{RHE} refers to the potential versus RHE.

Ultimately, the free energy of adsorbate at the given V_{RHE} and V_{SHE} is shown by Equation (4):

$$G_{ads} = G_{ads}^{PZC} + \mu \frac{C_H(V_{SHE} - V_{PZC})}{\varepsilon\varepsilon_0} - \frac{\alpha}{2}\left(\frac{C_H(V_{SHE} - V_{PZC})}{\varepsilon\varepsilon_0}\right)^2 - neV_{RHE} \quad (4)$$

The Cutoff charge density $\rho_{cut} = 0.0025$ Å^{-3}, and a surface tension parameter of 0.525 meV/Å^2.

For the relaxation freedom, Nørskov and colleagues [4] found that performing single-point calculations without a structural relaxation on transition metals may lead to misleading conclusions in the pH-field coupled analysis. In this work, all the electric field effect simulations and field-induced energetic calculations have considered full structural relaxations.

Solvation effects play a crucial role in electrochemical reactions occurring at the electrode-electrolyte interface [7]. Earlier studies have highlighted the pronounced solvent stabilization of HO* on Pt(111), presenting values ranging from 0.1 to 0.3 eV, contingent upon coverage[8]. Tripkovic et al. [9] identified a stabilization of HOO* by 0.5 eV, achieved through a semi-dissociated water layer on Pt(111). Meanwhile, Liu and colleagues [10] observed that O* and O_2* undergo negligible stabilization. In contrast, HO* and HOO* were found to be stabilized at approximately 0.6 eV and 0.7 eV, respectively. The Sb_2WO_6 synthesized in this study is categorized under metal oxides. Consequently, we utilized solvation correction energies of 0.15 eV for HO* and 0.4 eV for HOO*, as earlier determined on IrO_2 [7]."

3. Low-index surfaces of Sb_2WO_6 were selected for the analysis

We calculated the surface energies of set of high-index crystal facets (110), (011), and (101), and low-index crystal facets (100), (001), and (010) in **Table S5** and found that the energies of high-index crystal facets are higher than those of the low-index crystal facets. Therefore, we focused on the more stable (100), (001), and (010) crystal facets for further analysis, among which (100) has lowest surface energy value of -0.267 eV/Å^2. Next, we calculated surface Pourbaix diagram for (100), (001) and (010), as shown in **Figs. 5g** and **S11-S12**. In the ORR potential window, we observed that (001) is partly covered by O* at the high-potential region of ORR (**Fig. S12a-b**), while the catalyst surface of (010) was partly covered by HO* after the potential is higher than 0.75 V (*vs*. RHE) (**Fig. S12c-d**).

These conclusions are similar to the (100) surface (**Fig. 5g**). Furthermore, after developing the volcano model plotted with (100), (001), and (010) in **Fig. 5h**, (100) exhibited the highest activity. Because catalysis is site-specific (*i.e.*, the most active site/facet may predominant the overall activity of a catalyst) [11], we consider that the (100) facet is the origin of the high alkaline ORR activity of the Sb-W-O material. Considering the lowest surface energy and the highest identified activity, the (100) surface was mainly selected for the theoretical analysis. Besides, the lattices observed under TEM (Fig. 5d-e) are all orthogonal to (100), further suggesting that the exposed active surface are correctly modelled.

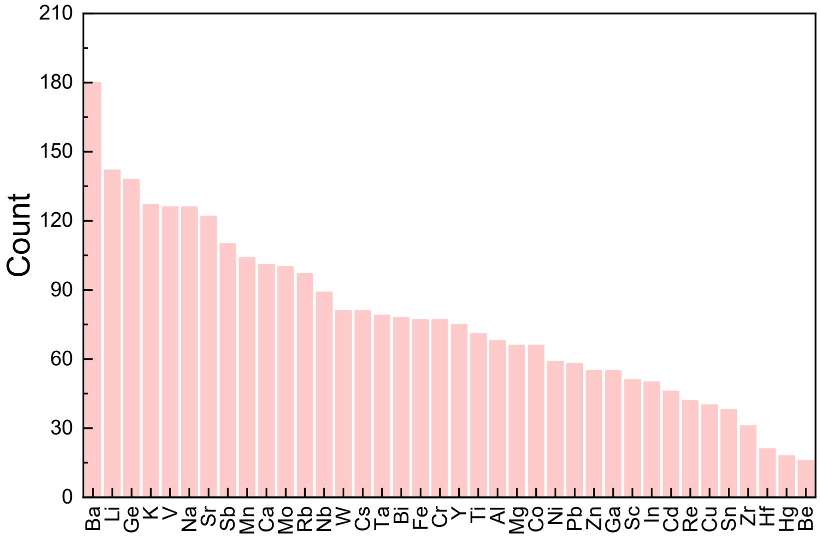

Fig. S1. The number of various elements present in our dataset.

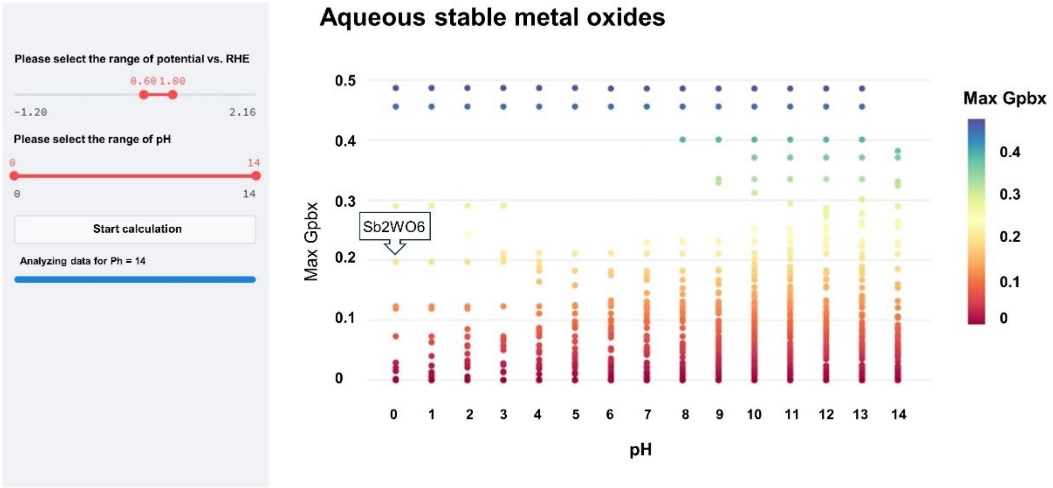

Fig. S2. Stable MOs for ORR across a pH range from 0 to 14 screened by the search engine developed in this work.

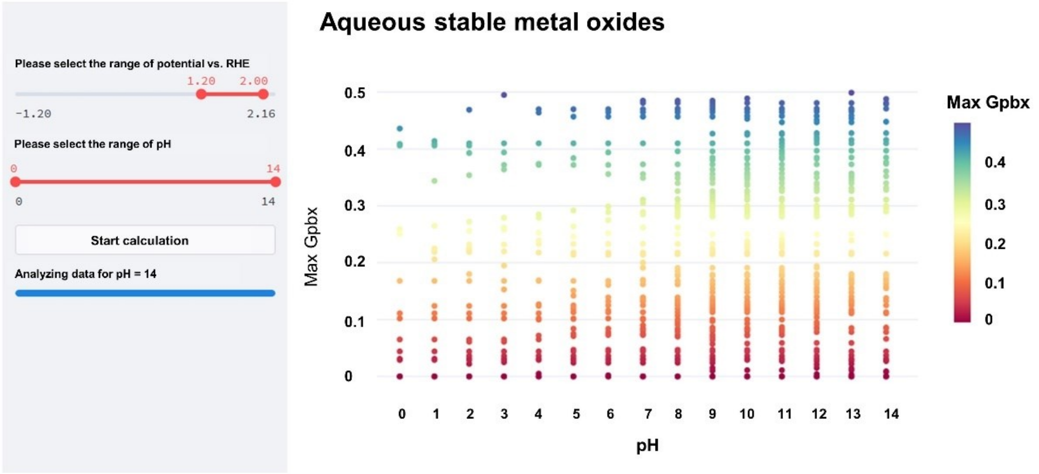

Fig. S3. Stable MOs for OER across a pH range from 0 to 14 screened by the search engine developed in this work.

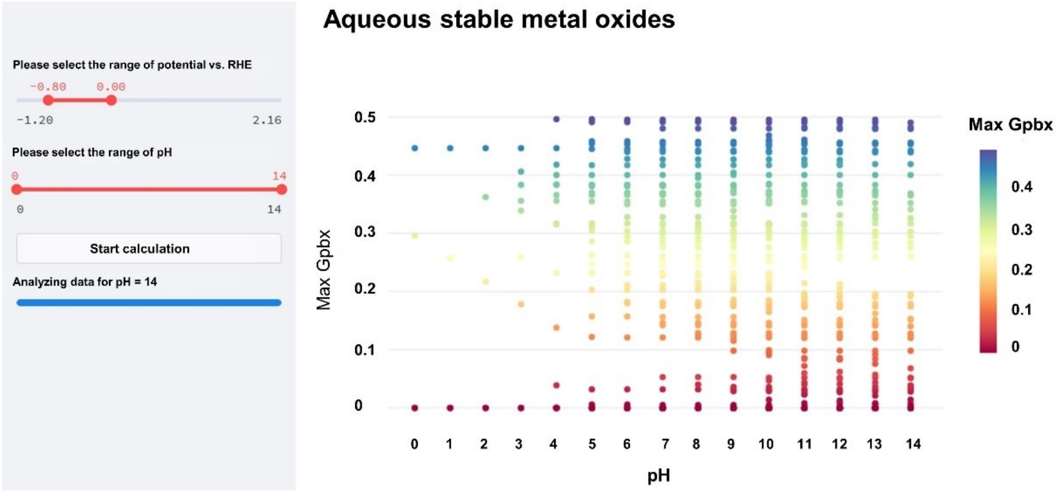

Fig. S4. Stable MOs for HER across a pH range from 0 to 14 screened by the search engine developed in this work.

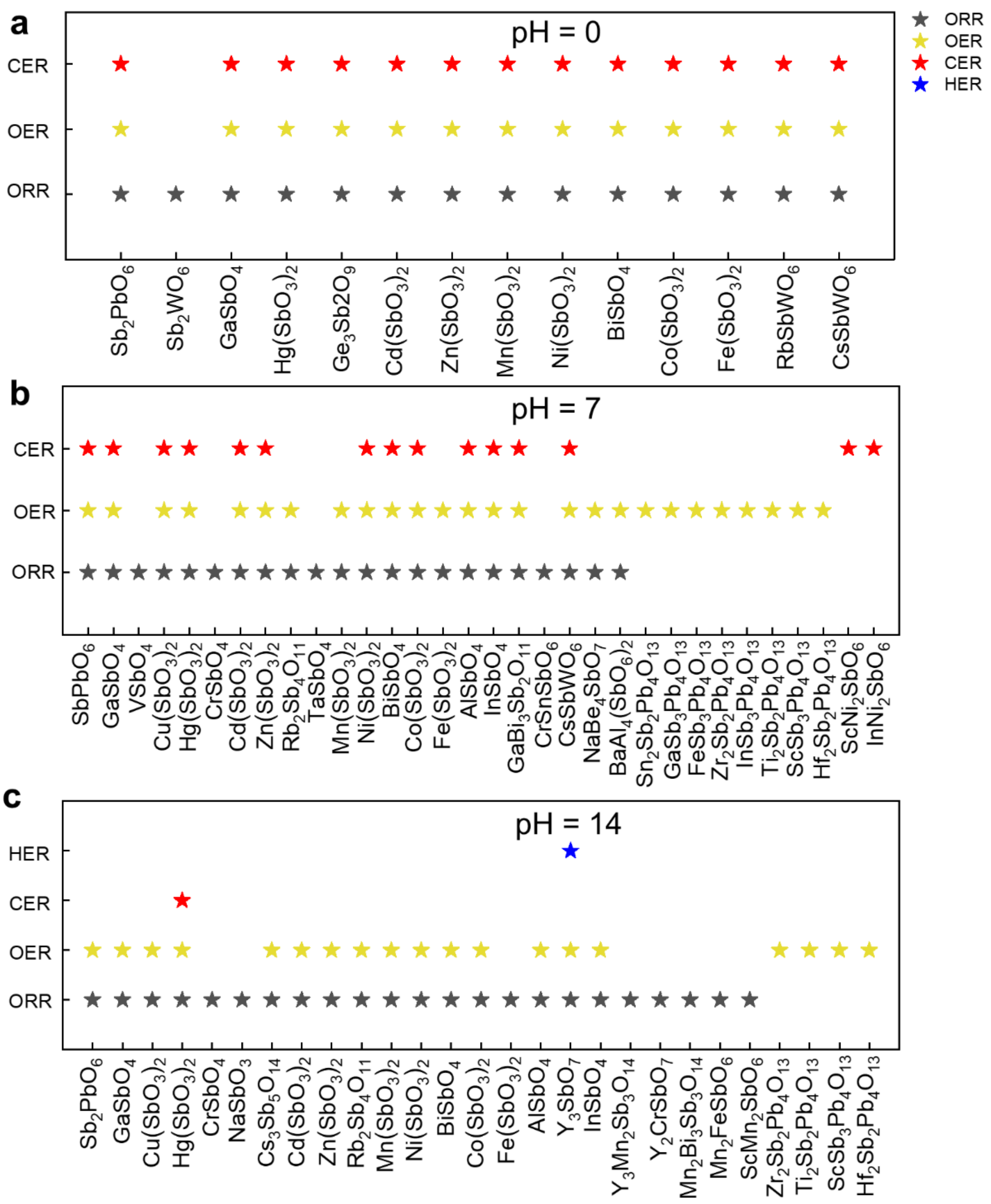

Fig. S5. Stable Sb-based materials under **a** pH = 0, **b** pH = 7, and **c** pH = 14 conditions for different reactions.

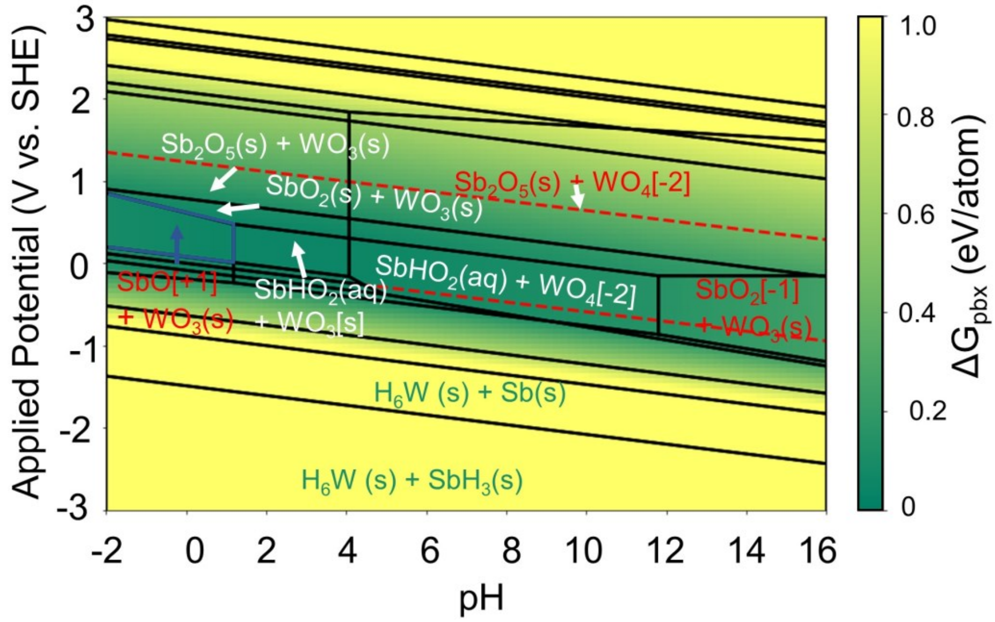

Fig. S6. Bulk Pourbaix diagram of Sb_2WO_6 extracted from Materials Project dataset.

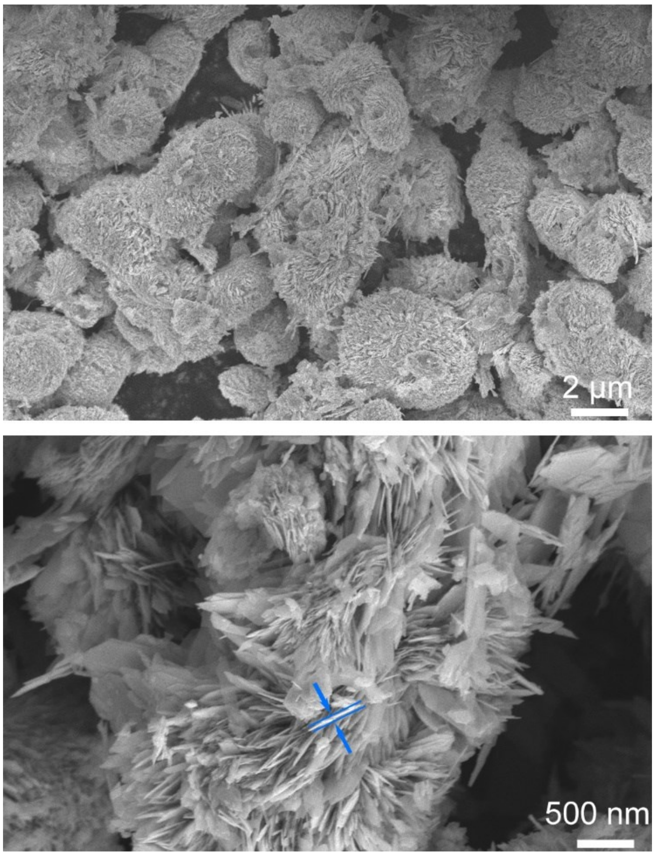

Fig. S7. SEM image of the as-synthesized Sb_2WO_6 nanoplates.

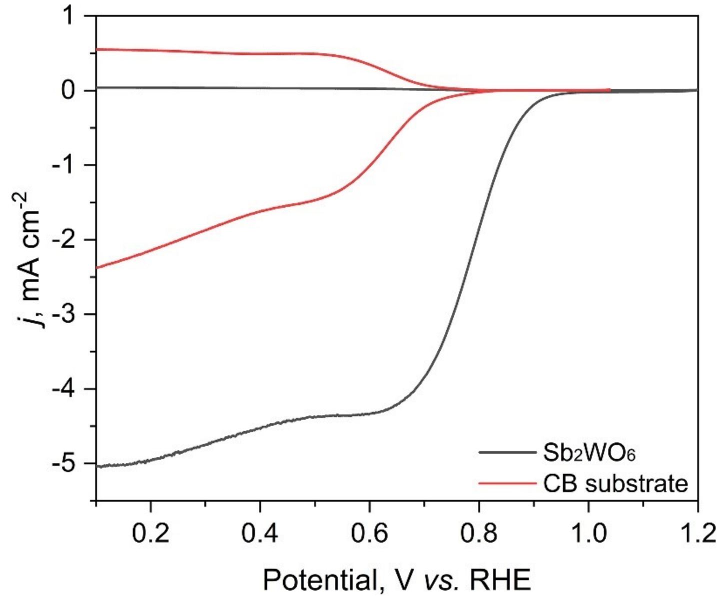

Fig. S8. ORR RRDE LSV curves of Sb_2WO_6 and blank carbon black (CB) substrate tested in an O_2-saturated 0.1 M KOH electrolyte at 1600 rpm, 25 °C. The CB loading is about 0.05 mg cm^{-2}, which is the same as that of CB loading during the test of Sb_2WO_6 sample (0.2 mg cm^{-2} of metal oxide + 0.05 mg cm^{-2} CB).

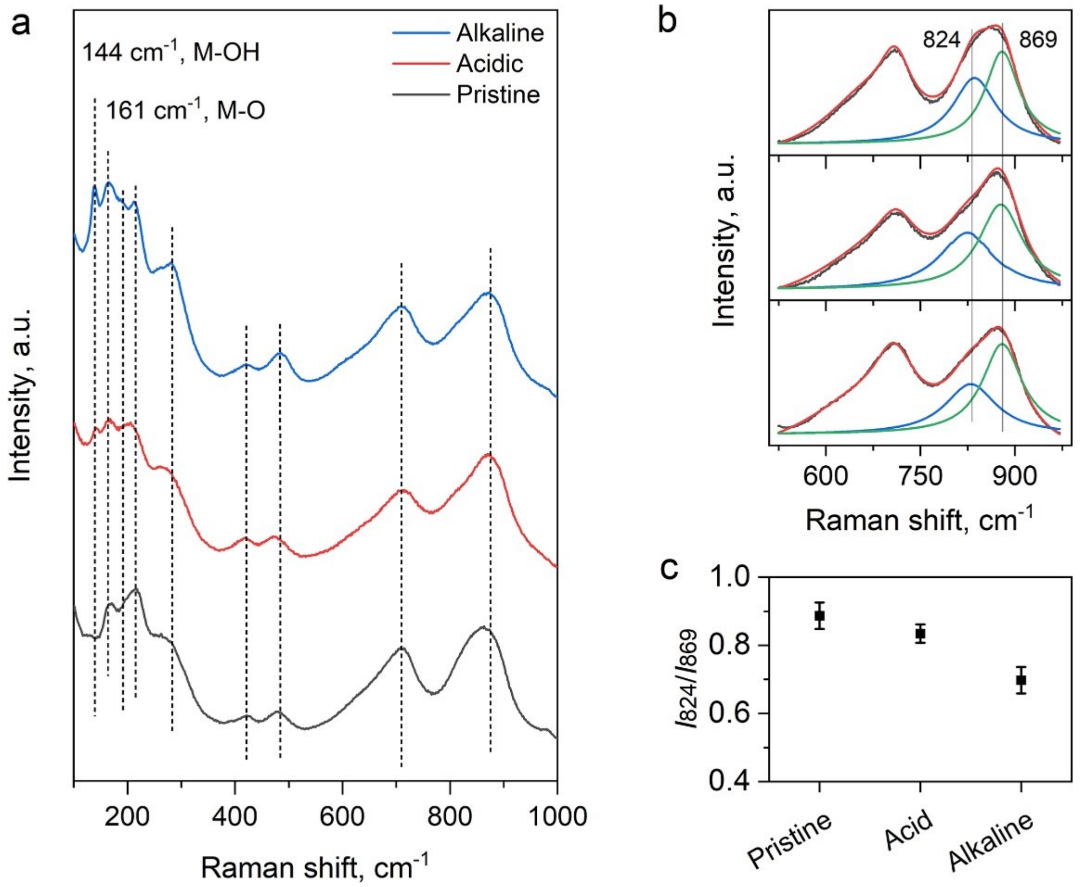

Fig. S9. a Raman spectra of the catalysts before and after stability test. **b** Fitting of the broad peak at 874 cm^{-1} with two Lorenzt peaks. **c** Relative intensity between the two fitted peaks.

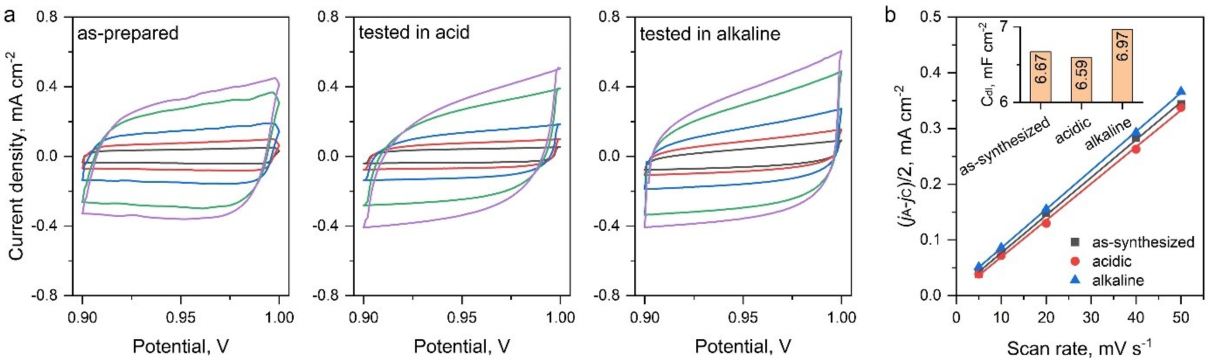

Fig. S10. ECSA measurement of the catalysts before and after stability test in different electrolytes. **a** CV curves obtained at different scan rates in a 0.1 M $HClO_4$ electrolyte. **b** Fitting current differences and scan rates of different catalysts. Inset compares the calculated C_{dl} of different catalysts.

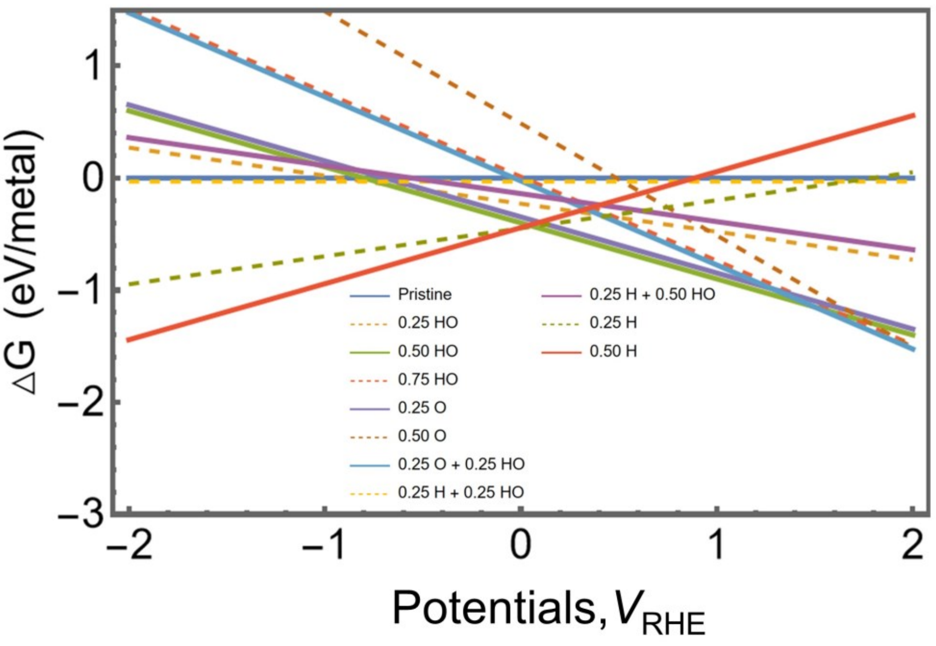

Fig. S11. Calculated 1D surface Pourbaix diagram of (100) crystal facets of Sb_2WO_6.

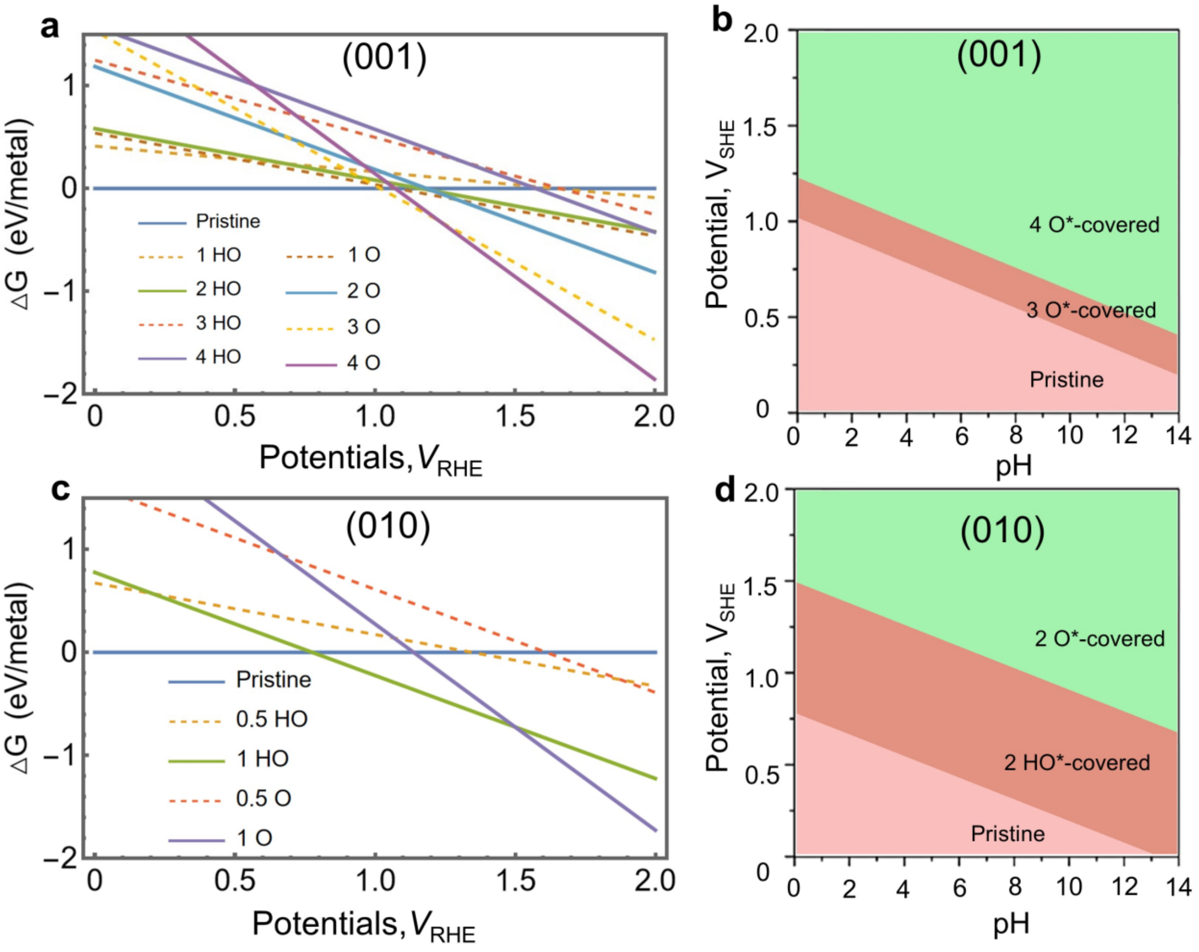

Fig. S12. Calculated 1D and 2D surface Pourbaix diagram of (**a-b**) (001) and (**c-d**) (010) crystal facets of Sb_2WO_6.

Table S1 Statistics of the metal elements from the defined stable MOs at a pH = 0. The normalized number is calculated by dividing the number of each element in the defined stable MOs by the number of various elements in our dataset.

	Number of various elements in our dataset	The number of each element in defined stable MOs for different reactions					The Normalized number of each element in defined stable MOs for different reactions				
		ORR	OER	CER	HER	NRR	ORR	OER	CER	HER	NRR
**Sb**	110	15	14	14	0	0	13.6	12.7	12.7	0.0	0.0
**W**	81	4	3	3	0	0	4.9	3.7	3.7	0.0	0.0
**Fe**	77	3	2	2	1	1	3.9	2.6	2.6	1.3	1.3
**Cr**	77	2	1	1	2	2	2.6	1.3	1.3	2.6	2.6
**Mo**	100	3	2	2	0	0	3.0	2.0	2.0	0.0	0.0
**Ge**	138	2	2	2	0	0	1.4	1.4	1.4	0.0	0.0
**Mg**	66	1	1	1	1	1	1.5	1.5	1.5	1.5	1.5
**Bi**	78	1	2	2	0	0	1.3	2.6	2.6	0.0	0.0
**Ti**	71	2	1	1	1	0	2.8	1.4	1.4	1.4	0.0
**Ni**	59	1	2	2	0	0	1.7	3.4	3.4	0.0	0.0
**Sn**	38	2	1	1	0	0	5.3	2.6	2.6	0.0	0.0
**Li**	142	1	0	0	1	1	0.7	0.0	0.0	0.7	0.7
**Co**	66	1	1	1	0	0	1.5	1.5	1.5	0.0	0.0
**Sc**	51	1	1	1	0	0	2.0	2.0	2.0	0.0	0.0
**Pb**	58	1	1	1	0	0	1.7	1.7	1.7	0.0	0.0
**Rb**	97	1	1	1	0	0	1.0	1.0	1.0	0.0	0.0
**Zn**	55	1	1	1	0	0	1.8	1.8	1.8	0.0	0.0
**Hg**	18	1	1	1	0	0	5.6	5.6	5.6	0.0	0.0
**Cs**	81	1	1	1	0	0	1.2	1.2	1.2	0.0	0.0
**Cd**	46	1	1	1	0	0	2.2	2.2	2.2	0.0	0.0
**Ga**	55	1	1	1	0	0	1.8	1.8	1.8	0.0	0.0
**Mn**	104	1	1	1	0	0	1.0	1.0	1.0	0.0	0.0
**Nb**	89	0	0	0	1	0	0.0	0.0	0.0	1.1	0.0

Table S2 Statistics of the metal elements from the defined stable MOs at a pH = 7. The normalized number is calculated by dividing the number of each element in the defined stable MOs by the number of various elements in our dataset.

	Number of various elements in our dataset	The number of each element in defined stable MOs for different reactions					The Normalized number of each element in defined stable MOs for different reactions				
		ORR	OER	CER	HER	NRR	ORR	OER	CER	HER	NRR
**Sb**	110	23	27	15	0	0	20.9	24.5	13.6	0.0	0.0
**Bi**	78	11	15	6	3	0	14.1	19.2	7.7	3.8	0.0
**Al**	68	11	9	3	11	0	16.2	13.2	4.4	16.2	0.0
**Sc**	51	10	9	6	7	0	19.6	17.6	11.8	13.7	0.0
**Ga**	55	10	11	3	3	0	18.2	20.0	5.5	5.5	0.0
**Ta**	79	14	0	0	11	0	17.7	0.0	0.0	13.9	0.0
**Cr**	77	11	1	1	8	2	14.3	1.3	1.3	10.4	2.6
**Nb**	89	6	6	4	6	0	6.7	6.7	4.5	6.7	0.0
**Ti**	71	6	7	1	8	0	8.5	9.9	1.4	11.3	0.0
**Cu**	40	10	6	3	2	0	25.0	15.0	7.5	5.0	0.0
**Fe**	77	9	3	0	6	1	11.7	3.9	0.0	7.8	1.3
**Co**	66	7	8	1	3	0	10.6	12.1	1.5	4.5	0.0
**In**	50	6	6	4	1	0	12.0	12.0	8.0	2.0	0.0
**Be**	16	5	5	2	4	1	31.3	31.3	12.5	25.0	6.3
**Pb**	58	1	13	2	0	0	1.7	22.4	3.4	0.0	0.0
**Sn**	38	7	4	3	2	0	18.4	10.5	7.9	5.3	0.0
**Zn**	55	5	4	2	3	0	9.1	7.3	3.6	5.5	0.0
**Ni**	59	2	4	5	1	0	3.4	6.8	8.5	1.7	0.0
**Cd**	46	3	2	3	2	0	6.5	4.3	6.5	4.3	0.0
**Zr**	31	2	3	1	2	0	6.5	9.7	3.2	6.5	0.0
**Mo**	100	3	2	1	1	0	3.0	2.0	1.0	1.0	0.0
**Mn**	104	4	3	0	0	0	3.8	2.9	0.0	0.0	0.0
**Hf**	21	1	3	1	1	1	4.8	14.3	4.8	4.8	4.8
**Mg**	66	1	2	2	1	1	1.5	3.0	3.0	1.5	1.5
**Ge**	138	2	2	1	1	0	1.4	1.4	0.7	0.7	0.0
**Y**	75	1	2	1	1	0	1.3	2.7	1.3	1.3	0.0
**V**	126	3	0	0	2	0	2.4	0.0	0.0	1.6	0.0
**Hg**	18	1	1	1	0	0	5.6	5.6	5.6	0.0	0.0
**Li**	142	1	0	0	1	1	0.7	0.0	0.0	0.7	0.7
**Cs**	81	1	1	1	0	0	1.2	1.2	1.2	0.0	0.0
**W**	81	1	1	1	0	0	1.2	1.2	1.2	0.0	0.0
**Na**	126	1	1	0	0	0	0.8	0.8	0.0	0.0	0.0
**Rb**	97	1	1	0	0	0	1.0	1.0	0.0	0.0	0.0
**Ba**	180	1	1	0	0	0	0.6	0.6	0.0	0.0	0.0
**Ca**	101	1	0	0	0	0	1.0	0.0	0.0	0.0	0.0

Table S3 Statistics of the metal elements from the defined stable MOs at a pH = 14. The normalized number is calculated by dividing the number of each element in the defined stable MOs by the number of various elements in our dataset.

	Number of various elements in our dataset	The number of each element in defined stable MOs for different reactions					The Normalized number of each element in defined stable MOs for different reactions				
		ORR	OER	CER	HER	NRR	ORR	OER	CER	HER	NRR
**Sb**	110	24	20	1	1	0	21.8	18.2	0.9	0.9	0.0
**Y**	75	17	14	0	12	0	22.7	18.7	0.0	16.0	0.0
**Ta**	79	16	1	0	19	0	20.3	1.3	0.0	24.1	0.0
**Ti**	71	10	9	0	10	0	14.1	12.7	0.0	14.1	0.0
**Cr**	77	14	1	0	11	2	18.2	1.3	0.0	14.3	2.6
**Mn**	104	21	3	0	3	0	20.2	2.9	0.0	2.9	0.0
**Bi**	78	10	7	2	5	0	12.8	9.0	2.6	6.4	0.0
**Fe**	77	13	3	0	5	1	16.9	3.9	0.0	6.5	1.3
**Sc**	51	6	5	1	5	0	11.8	9.8	2.0	9.8	0.0
**Mg**	66	4	5	1	3	1	6.1	7.6	1.5	4.5	1.5
**Cd**	46	6	5	0	3	0	13.0	10.9	0.0	6.5	0.0
**Co**	66	3	8	0	1	0	4.5	12.1	0.0	1.5	0.0
**Pb**	58	2	9	0	1	0	3.4	15.5	0.0	1.7	0.0
**Cu**	40	4	7	0	1	0	10.0	17.5	0.0	2.5	0.0
**Zr**	31	3	4	2	3	0	9.7	12.9	6.5	9.7	0.0
**Ni**	59	3	5	1	0	0	5.1	8.5	1.7	0.0	0.0
**Hf**	21	2	3	1	2	1	9.5	14.3	4.8	9.5	4.8
**Zn**	55	4	2	0	1	0	7.3	3.6	0.0	1.8	0.0
**Li**	142	4	0	0	1	1	2.8	0.0	0.0	0.7	0.7
**Ba**	180	3	0	0	3	0	1.7	0.0	0.0	1.7	0.0
**Ca**	101	2	0	0	2	0	2.0	0.0	0.0	2.0	0.0
**Sn**	38	2	1	0	1	0	5.3	2.6	0.0	2.6	0.0
**In**	50	2	1	0	1	0	4.0	2.0	0.0	2.0	0.0
**Nb**	89	1	1	0	1	0	1.1	1.1	0.0	1.1	0.0
**W**	81	1	1	0	1	0	1.2	1.2	0.0	1.2	0.0
**Hg**	18	1	1	1	0	0	5.6	5.6	5.6	0.0	0.0
**Na**	126	2	0	0	1	0	1.6	0.0	0.0	0.8	0.0
**V**	126	0	0	0	3	0	0.0	0.0	0.0	2.4	0.0
**Cs**	81	1	1	0	0	0	1.2	1.2	0.0	0.0	0.0
**K**	127	1	0	0	1	0	0.8	0.0	0.0	0.8	0.0
**Al**	68	1	1	0	0	0	1.5	1.5	0.0	0.0	0.0
**Ga**	55	1	1	0	0	0	1.8	1.8	0.0	0.0	0.0
**Rb**	97	1	1	0	0	0	1.0	1.0	0.0	0.0	0.0
**Ge**	138	1	0	0	0	0	0.7	0.0	0.0	0.0	0.0
**Sr**	122	0	0	0	1	0	0.0	0.0	0.0	0.8	0.0

Table S4. XPS peak position assignment of different elements.

Peak position, eV	Assignment
34.28	Sb 4$d_{5/2}$
35.58	Sb 4$d_{3/2}$
35.88	W 4$f_{7/2}$
37.88	W 4$f_{5/2}$
530.08	Sb 3$d_{5/2}$
531.48	O 1*s* (in metal oxides)
539.38	Sb 3$d_{3/2}$

Table S5. Surface energies of different surfaces of Sb_2WO_6 (eV Å^{-2}).

Surface	100-1	100-2	010-1	010-2	001-1	001-2	110	011	101
Surface energy	–0.221	–0.267	–0.218	–0.214	–0.153	–0.211	-0.13	-0.16	-0.17

Table S6. Field effects on the Sb_2WO_6

	O_2*		HOO*		O*		HO*		H_2O_2*	
	α	μ	α	μ	α	μ	α	μ	α	μ
Sb_2WO_6	-0.07	0.38	0.129	0.153	-0.06	0.03	0.09	0.10	0.334	0.06

Reference

[1] Z. Wang, Y.-R. Zheng, I. Chorkendorff, J. K. Nørskov, *ACS Energy Lett.* **2020**, *5*, 2905.

[2] P. Giannozzi, S. Baroni, N. Bonini, M. Calandra, R. Car, C. Cavazzoni, D. Ceresoli, G. L. Chiarotti, M. Cococcioni, I. Dabo, A. Dal Corso, S. de Gironcoli, S. Fabris, G. Fratesi, R. Gebauer, U. Gerstmann, C. Gougoussis, A. Kokalj, M. Lazzeri, L. Martin-Samos, N. Marzari, F. Mauri, R. Mazzarello, S. Paolini, A. Pasquarello, L. Paulatto, C. Sbraccia, S. Scandolo, G. Sclauzero, A. P. Seitsonen, A. Smogunov, P. Umari, R. M. Wentzcovitch, *J. Phys. Condens. Matter.* **2009**, *21*, 395502.

[3] L. Fumagalli, A. Esfandiar, R. Fabregas, S. Hu, P. Ares, A. Janardanan, Q. Yang, B. Radha, T. Taniguchi, K. Watanabe, G. Gomila, K. S. Novoselov, A. K. Geim, *Science* **2018**, *360*, 1339.

[4] S. R. Kelly, C. Kirk, K. Chan, J. K. Nørskov, *J. Phys. Chem. C* **2020**, *124*, 14581.

[5] a) Z. Duan, G. Henkelman, *ACS Catal.* **2019**, *9*, 5567; b) X. Hu, S. Chen, L. Chen, Y. Tian, S. Yao, Z. Lu, X. Zhang, Z. Zhou, *J. Am. Chem. Soc.* **2022**, 144, 18144–18152.

[6] a) R. Sundararaman, K. Letchworth-Weaver, K. A. Schwarz, *J. Chem. Phys.* **2018**, *148*, 144105; b) N. G. Hörmann, O. Andreussi, N. Marzari, *J. Chem. Phys.* **2019**, *150*, 041730.

[7] J. A. Gauthier, C. F. Dickens, L. D. Chen, A. D. Doyle, J. K. Nørskov, *J. Phys. Chem. C* **2017**, *121*, 11455.

[8] J. K. Nørskov, J. Rossmeisl, A. Logadottir, L. Lindqvist, J. R. Kitchin, T. Bligaard, H. Jónsson, *The J. Phys. Chem. B* **2004**, *108*, 17886.

[9] V. Tripkovic, E. Skúlason, S. Siahrostami, J. K. Nørskov, J. Rossmeisl, *Electrochim. Acta* **2010**, *55*, 7975.

[10] S. Liu, M. G. White, P. Liu, *J. Phys. Chem. C* **2016**, *120*, 15288.

[11] J. K. Nørskov, T. Bligaard, B. Hvolbæk, F. Abild-Pedersen, I. Chorkendorff, C. H. Christensen, *Chem. Soc. Rev.* **2008**, *37*, 2163.
